# Multicentre Italian study of SARS-CoV-2 infection in children and adolescents, preliminary data as at 10 April 2020

**DOI:** 10.2807/1560-7917.ES.2020.25.18.2000600

**Published:** 2020-05-07

**Authors:** Silvia Garazzino, Carlotta Montagnani, Daniele Donà, Antonella Meini, Enrico Felici, Gianluca Vergine, Stefania Bernardi, Roberta Giacchero, Andrea Lo Vecchio, Paola Marchisio, Giangiacomo Nicolini, Luca Pierantoni, Ivana Rabbone, Giuseppe Banderali, Marco Denina, Elisabetta Venturini, Andrzej Krzysztofiak, Raffaele Badolato, Sonia Bianchini, Luisa Galli, Alberto Villani, Guido Castelli-Gattinara, the Italian SITIP-SIP Pediatric Infection Study Group

**Affiliations:** 1 Paediatric Infectious Diseases Unit, Regina Margherita Children’s Hospital, University of Turin, Turin, Italy; 2 Infection Disease Unit, Meyer Children’s University Hospital, Florence, Italy; 3 Division of Paediatric Infectious Diseases, Department for Woman and Child Health, University of Padua, Padua, Italy; 4 Pediatrics Clinic, University of Brescia and ASST-Spedali Civili of Brescia,, Brescia, Italy; 5 Paediatric and Pediatric Emergency Unit, The Children Hospital, AO SS Antonio e Biagio e Cesare Arrigo, Alessandria, Italy; 6 UOC Pediatria, Ospedale degli Infermi di Rimini, Rimini, Italy; 7 Universitarian-Hospital Department, Ospedale Bambino Gesù IRCCS, Rome, Italy; 8 UOC Pediatria e Patologia Neonatale -ASST Lodi, Lodi, Italy; 9 Department of Translational Medical Science, Section of Paediatrics, University of Naples Federico II, Naples, Italy; 10 Fondazione IRCCS Cà Granda Ospedale Maggiore Policlinico, Milan, Italy; 11 UOC Pediatria, San Martino Hospital, Belluno, Italy; 12 Paediatric Emergency Unit, Policlinico di Sant’Orsola, Bologna, Italy; 13 Division of Paediatrics, Department of Health Sciences, University of Piemonte Orientale, Novara, Italy; 14 Department of Paediatrics, ASST Santi Paolo e Carlo, University of Milan, Milan, Italy; 15 The members of the Italian SITIP-SIP SARS-CoV-2 paediatric infection study group are listed at the end of this article

**Keywords:** SARS-CoV-2 infection, covid-19, children, pneumonia, hydroxychloroquine

## Abstract

Data on features of severe acute respiratory syndrome coronavirus 2 (SARS-CoV-2) in children and adolescents are scarce. We report preliminary results of an Italian multicentre study comprising 168 laboratory-confirmed paediatric cases (median: 2.3 years, range: 1 day–17.7 years, 55.9% males), of which 67.9% were hospitalised and 19.6% had comorbidities. Fever was the most common symptom, gastrointestinal manifestations were frequent; two children required intensive care, five had seizures, 49 received experimental treatments and all recovered.

Since the end of December 2019, coronavirus disease (COVID-19) caused by severe acute respiratory syndrome coronavirus 2 (SARS-CoV-2) has rapidly spread worldwide becoming the first pandemic of the 21st century. Despite the high number of people affected, data on clinical features and prognostic factors in children and adolescents are limited.

We report the preliminary results of a national multicentre study, promoted by the Italian Society of Paediatric Infectious Diseases (SITIP), within the Italian Society of Paediatrics (SIP). The study investigates epidemiological, clinical and therapeutic aspects of SARS-CoV-2 infection in infants, children and adolescents, hereafter referred to as paediatric population or children.

## Participating physicians, hospitals and patients

The multicentre study involves 11 of 13 exclusively paediatric hospitals and 51 of 390 paediatric units across Italy, but predominantly in central and northern regions. Of approximately 15,900 paediatricians working in the national health system, more than 10,000 are members of SIP. Retrospective data collection started on 25 March 2020.

The presented data include all paediatric patients in whom COVID-19 was documented by at least one nasal/pharyngeal swab specimen positive for SARS-CoV-2 nucleic acid using real-time reverse-transcriptase polymerase-chain-reaction (RT-PCR) assay.

Ethical approval of the ethical committee of the coordinating Centre in Turin (Comitato Etico Interaziendale AOU Città della Salute e della Scienza di Torino – AO Ordine Mauriziano di Torino – ASL Città di Torino) was provided on 24 March 2020, protocol number 0031296. Data collection was allowed by written consent of at least one parent; patients’ data were de-identified.

## Findings

As at 10 April 2020, data for 168 children aged 1 day to 17 years, 94 (55.9%) males and 74 (40.1%) females, with confirmed COVID-19 and an adequate follow-up were available. Adequate follow-up was the period considered necessary by the clinician to define the final outcome, in most instances at least 2 weeks. The mean age was 5 years (median: 2.3 years, interquartile range (IQR): 0.3–9.6 years); 15 were neonates (Table). The majority of children (65.1%) were hospitalised: of these, only 17 (15.5%) were referred to hospital after seeing a paediatrician or family doctor. Hospital admission was inversely related to age (p < 0.01; Fisher exact test); among infected children under 1 year of age, 52/66 were hospitalised vs 24/38, 13/24 and 21/40 among the 1 to 5 year-olds, 6 to 10 year-olds and over 10 year-olds, respectively.

**Table:** Characteristics of SARS-CoV-2-infected children, Italy, as at 10 April 2020 (n = 168)

Parameter	Total (n)	Percentage (%)
**Age (years)**		
Mean, median (IQR)	5, 2.3 (0.3–9.6)	NA
**Age groups**		
< 1 year^a^	66	39.3
1–5 years	38	22.6
6–10 years	24	14.3
11–17 years	40	23.8
**Sex**		
Males	94	55.9
Females	74	44.1
**Signs and symptoms^b^**		
Fever ranging from 37.5–39°C	138	82.1
Cough	82	48.8
Rhinitis	45	26.8
Diarrhoea	22	13.1
Dyspnoea	16	9.5
Pharyngitis	9	5.4
Vomiting	9	5.4
Conjunctivitis	6	3.6
Chest pain	4	2.4
Fatigue	3	1.8
Non-febrile seizures	3	1.8
Febrile seizures	2	1.2
**Hospital admission within age groups**		
< 1 year	52	78.8
1–5 years	24	63.2
6–10 years	13	54.2
11–17 years	21	52.5
Total	110	65.1

IQR: interquartile range; NA: not applicable; SARS-CoV-2: severe acute respiratory syndrome coronavirus 2.

^a^ Of coronavirus disease (COVID-19) cases in this age group, 15 of 66 were less than 4 weeks of age.

^b^ Several answers were possible.

Thirty-three children (19.6%) had underlying chronic diseases, such as chronic lung disease (n = 7), congenital malformations or complex genetic syndromes (n = 14), cancer (n = 4), epilepsy (n = 5), gastrointestinal (n = 2) or metabolic disorders (n = 1) and seven were immunosuppressed (n = 4) or immunocompromised (n = 3). The hospitalisation rate was similar between children with comorbidities and those without (23/33 vs 87/135, respectively; p = 0.68, Fisher exact test).

Close contact with a COVID-19 infected person outside the family was rarely reported; conversely, 67.3% (113/168) of children had at least one parent who tested positive for SARS-CoV-2 infection. Symptom onset in relatives frequently (88/113, 77.8%) preceded symptoms in the infected child between 1 to 14 days.

All but four (2.5%) enrolled children were symptomatic. Fever ranging from 37.5 to 39 °C was the most common symptom (82.1%), followed by cough (48.8%) and rhinitis (26.8%). Interestingly, 31 children (18.4%) developed gastrointestinal symptoms (vomiting and/or diarrhoea), while five had seizures; of these, three children had a known history of epilepsy, one child had a past history of febrile seizures and the remaining one had a first episode of febrile seizures as onset of COVID-19e and SARS-CoV-2 encephalitis was ruled out. The mean interval between symptom onset and first medical evaluation was 1.6 days (range: 0–18).

In children who underwent blood investigations, the increase of C-reactive protein above 0.5 mg/dl was the most common finding (47/121, 38.8%), while other alterations frequently encountered in adults, such as leukopenia, neutropenia, lymphopenia, increased CK or LDH values, were rare (data not shown).

### Complications and co-infections

Thirty-three children (19.6%) developed complications, such as interstitial pneumonia (n = 26), severe acute respiratory illness (n = 14) and peripheral vasculitis (n = 1); two of the 33, a preterm neonate and a 2-month-old infant with congenital heart disease, required intensive care unit (ICU) admission and treatment with mechanical ventilation. Non-invasive oxygen-treatment was administered to 16 of 168 (9.5%) children. No child underwent chest computed tomography scan; pneumonia was assessed either by X-ray or ultrasound in 75 of the children.

A viral co-infection was documented in 10 children (5.9%), including three respiratory syncytial virus, three rhinovirus, two Epstein-Barr virus, one influenza A virus and one non-SARS coronavirus infection. A bacterial co-infection with *Streptococcus pneumoniae* was also documented.

### Antiviral treatment

Experimental treatments for SARS-CoV-2 infection, including lopinavir/ritonavir (lopinavir component: 230 mg/m^2^ of body surface area twice daily), hydroxychloroquine (2.5 mg/kg twice daily) and/or azithromycin (10 mg/kg once daily)/clarithromycin (7.5 mg/kg twice daily), were administered to 49 children (29.2%). A systemic steroid was administered only in one case. Antiviral treatments were preferentially given to children who were more severely ill (data not shown).

## Discussion

SARS-CoV-2 infection in children differs from adult disease with respect to clinical manifestations and outcome. Our data confirmed that case fatality in children is very low: only a few fatal COVID cases have been reported in the literature thus far [1-3]. In our series, all children, including those with comorbidities, recovered fully, and no sequelae were reported at the time of submission.

Italy has been among of the countries most affected by COVID-19, with more than 140,000 infected cases and around 17,000 deaths as at 10 April 2020 [4]. The number of cases and case-fatality rate in Italian adults with COVID-19 are higher compared with many other countries [5]. This may be because of an older mean population age, higher frequency of comorbidities in the older population, and the limited number of rhino-pharyngeal swabs performed on asymptomatic people during the initial phase of the Italian epidemic. In this scenario, data from our paediatric multicentre study confirm the different course of the infection in the paediatric age group: children were a marginal percentage of the Italian infected population admitted to hospital and tended to develop benign, pauci-symptomatic disease.

The contribution of children to disease transmission is still under debate, including whether they might serve as facilitators of viral transmission, being a silent reservoir for the virus. Many hypotheses have been formulated on the mechanisms underlying children’s lower susceptibility to severe SARS-CoV-2 infection compared with adults; these include an immature receptor system, specific regulatory mechanisms in the immune respiratory system and cross-protection by antibodies directed towards common viral infections in infancy [6]. However, nearly 40% of the children included in this report were under 1 year of age and the majority of them were hospitalised, suggesting a higher susceptibility to symptomatic COVID-19 in this specific age group: the two children who required ICU admission were a neonate and a 2-month-old infant. However, the high number of children under 1 year of age in our study may also reflect both a higher tendency for families to seek medical advice for younger children and a higher propensity among clinicians to admit them to hospitals. Also in the United States (US), hospitalisation was more common among children under 1 year of age than in other paediatric age groups, including ICU admission [1]. According to the Italian national public health institute’s surveillance report of 10 April, SARS-CoV-2 infection affected a total of 1,936 children, of whom 5.2% were hospitalised; the percentage of hospitalised children within the 0 to 1-year-old age group was 10.9%. A rough estimate of the general hospitalisation rate in the Italian paediatric population is 39.6 per 1,000 children [7].

Similar to what was reported in paediatric studies from China and the US, we observed a slightly higher, although not statistically significant, prevalence in males in all age groups (data not shown), supporting the hypothesis that sex-linked genetic factors may influence susceptibility to COVID-19.

Fever was the most common encountered symptom in our cohort: this is in contrast with data reported in Chinese and US American children in whom fever was less common (36–56%) compared with cough or pharyngitis [1,2,8-10]. Conversely, proportions of gastrointestinal symptoms were similar among the three cohorts, ranging from 6.4 to 11% for nausea and vomiting and from 8.8 to 13% for diarrhoea [1,2,8-10]. Neurological manifestations, consisting in febrile and non-febrile seizures, were observed in 3% of children at onset of COVID-19, although none developed SARS-CoV-2-related encephalitis.

Although only preliminary data are presented, our study has several limitations. First, our population includes children and adolescents under 18 years of age: this make some results difficult to compare with other publications that consider children and adolescents up to 15 or 16 years. Secondly, the limited sample size for some analyses does not allow to draw definite conclusions. For example, because of small numbers and differences in demographic conditions between children who did vs did not receive antiviral treatments, clinical progression of treated and untreated children could not be compared. Also, with a wider population, specific comparison and analysis in different age groups should be looked specifically at.

Despite these limitations, this is to our knowledge the largest cohort on the characteristics of laboratory-confirmed COVID-19 in European children. At present, most of the paediatric data are from Chinese studies; of these, many also included children without a laboratory diagnosis and in them, the disease seems to have taken a more severe course than in children with laboratory-confirmed disease [8]. According to some authors, this may be because a number of children improperly categorised as having COVID-19 might have been infected by other aggressive pathogens [11].

In conclusion, our findings show a favourable clinical course of COVID-19 infection in children and adolescents in Italy, where the case-fatality rates observed in adults seem high compared with several other countries. Consequently, the diagnostic, clinical and even therapeutic approach in children might be more conservative than in adults, for example reserving chest computed tomography scan, hospital admission and antiviral treatments (unless more effective and safe drugs will become available) to selected situations.

## The Italian SITIP-SIP SARS-CoV-2 paediatric infection study group

Filippo Salvini, Laura Abbagnato, Elio Castagnola, Icilio Dodi, Cesare Ghitti, Paola Lippi, Rino Agostiniani, Simonetta Cherubini, Piero Valentini, Paola Gianino, Angelina Vaccaro, Paolo Manzoni, Paola Verna, Pasquale Comberiati, Paola Di Filippo, Paola Gallia, Gianna Battezzati, Ludovica Fiore, Cristina Dalmazzo, Eleonora Tappi, Marta Lazzerini, Pier-Angelo Tovo, Carlo Scolfaro, Giulia Pruccoli, Ugo Ramenghi, Carlo Giaquinto, Liviana Da Dalt, Gianluca Tornese, Paola Berlese, Alessandro Plebani, Emma Concetta Manno, Veronica Santilli, Laura Lancella, Laura Cursi, Andrea Campana, Elena Bozzola, Samantha Bosis, Marcello Lanari, Carmine Pecoraro, Paolo Del Barba, Emanuele Nicastro, Susanna Esposito, Gian Vincenzo Zuccotti, Giovanni Corsello, Fabio Cardinale, Anna Maria Tocco, Giuseppina Ballardini, Carlo Agostoni, Elena Chiappini, Giuseppe Indolfi, Bussolini Anna, Salvatore Cazzato, Giorgio Zavarise, Claudio Pignata, Federico Marchetti.
