# Authors’ correction for Euro Surveill. 2020;25(18)

**DOI:** 10.2807/1560-7917.ES.2020.25.29.2007231

**Published:** 2020-07-23

**Authors:** Eurosurveillance editorial team

**Affiliations:** 1 European Centre for Disease Prevention and Control (ECDC), Stockholm, Sweden

In the article ‘Multicentre Italian study of SARS-CoV-2 infection in children and adolescents, preliminary data as at 10 April 2020’ by Garazzino et al. published on 7 May 2020, the first name of Luisa Abbagnato was corrected in the Italian SITIP-SIP SARS-CoV-2 paediatric infection study group on 20 July 2020, upon request of the authors.

